# Identification of key regulatory molecules in the early development stage of Alzheimer's disease

**DOI:** 10.1111/jcmm.18151

**Published:** 2024-03-01

**Authors:** Bin Huang, Guan‐yong Ou, Ni Zhang

**Affiliations:** ^1^ Clinical Laboratory Fifth Affiliated Hospital of Southern Medical University Guangzhou China; ^2^ School of Medicine Southern University of Science and Technology Shenzhen China; ^3^ Department of Physiology Shantou University Medical College Shantou China

**Keywords:** Alzheimer's disease, bioinformatics, biomarker, ceRNA, DEGs, lncRNA

## Abstract

Alzheimer's disease (AD) is one of the most common neurodegenerative diseases, the incidence of which increases with age, and the pathological changes in the brain are irreversible. Recent studies have highlighted the essential role of long noncoding RNAs (lncRNAs) in AD by acting as competing endogenous RNAs (ceRNAs). Our aim was to construct lncRNA‐associated ceRNA regulatory networks composed of potential biomarkers for the early stage of AD. AD related datasets come from AlzData and GEO databases. The R package ‘Limma’ identifies differentially expressed genes (DEGs), Kyoto Encyclopedia of Genes and Genomes (KEGG) and Gene Ontology (GO) databases for functional enrichment analysis. Protein–protein interactions (PPIs) in DEGs were constructed in the STRING database, and Cytoscape software identified DEGs. Convergent functional genomics (CFG) analysis of differentially expressed hub genes (referred to as early‐DEGs) in the brain before the development of AD pathology. The AlzData database analyses the expression levels of early‐DEGs in different nerve cells. The lncRNA‐miRNA‐mRNA regulatory network was established according to the ceRNA hypothesis. We identified four lncRNAs (XIST, NEAT1, KCNQ1OT1 and HCG18) and four miRNAs (hsa‐let‐7c‐5p, hsa‐miR‐107, hsa‐miR‐129‐2‐3p and hsa‐miR‐214‐3p) were preliminarily identified as potential biomarkers for early AD, competitively regulating *Atp6v0b*, *Atp6v1e1 Atp6v1f* and *Syt1*. This study indicates that NEAT1, XIST, HCG18 and KCNQ1OT1 act as ceRNAs in competitive binding with miRNAs to regulate the expression of *Atp6v0b*, *Atp6v1e1*, *Atp6v1f* and *Syt1* before the occurrence of pathological changes in AD.

## 1 INTRODUCTION

Alzheimer's disease (AD) is a progressive neurodegenerative disease characterized by cognitive decline. The disease has an unremitting course and its incidence increases with age.^1^ An estimated 40 million people are affected by AD worldwide, and it is the sixth leading cause of death.^2^, ^3^ β‐amyloid (Aβ) plaques and intracellular neurofibrillary tangles (NFTs) composed of hyperphosphorylated tau protein are the pathological hallmarks of AD. They tend to induce neuronal degeneration, neuroinflammation and glial cell activation, ultimately resulting in AD.^4^ The currently available pharmacological therapies for AD only provide symptom relief and there are no effective drugs to cure this disease.^5^ By the time a clinical diagnosis of AD is established, neurons in many brain regions have already undergone substantial changes. At the early stage of the disease, patients do not usually have symptoms (such as cognitive or functional decline), but pathological changes have already occurred in the nervous system. By the time of appearance of clinical manifestations of AD, the quality of life of patients and their families is severely impacted. Therefore, identifying effective biomarkers is critical to uncover the underlying mechanisms and facilitate clinical diagnosis and treatment of AD.

Long noncoding RNAs (lncRNAs) refer to RNAs exceeding 200 nucleotides (nt) in length that do not code for proteins. It is estimated that lncRNAs are more numerous than protein‐coding genes in humans.^6^ The development of more advanced RNA sequencing technologies has catalysed the advancement of epigenomics and computational prediction techniques.^7^, ^8^ Studies have demonstrated the involvement of lncRNAs in the causation of human neurological diseases such as schizophrenia, autism spectrum disorder (ASD), Parkinson's disease (PD), Huntington's disease (HD) and AD.^9^

Micro RNAs (miRNAs) are single‐stranded RNAs that act on the partially complementary site of the 3′‐untranslated region (UTR) of the target mRNA through the base pairing process and negatively regulate gene expression, resulting in translational inhibition or attenuation of the target gene.^10^ Competing endogenous RNAs (ceRNAs) can communicate and regulate each other by specifically competing with miRNAs for shared mRNAs.^11^, ^12^, ^13^ lncRNAs are also a type of ceRNAs involved in this process.^14^ Studies have consistently reported the link between lncRNAs and AD. Among them, BACE1‐as is the most widely studied lncRNA. The expression level of BACE1 in plasma was identified as a potential biomarker of brain amyloidosis in AD patients.^15^, ^16^ BACE1‐as was shown to upregulate BACE1 mRNA and protein expression levels by preventing miRNA‐485‐5p from binding to the open reading frame of BACE1,^17^ resulting in enhanced Aβ amyloid formation and the reduced protective effects miR‐132‐3p overpression on synaptic plasticity.^18^ BC200 is a lncRNA that regulates dendritic neuroprotein synthesis and synaptic plasticity by targeting eukaryotic initiation factor 4A (eIF4A).^19^ Another lncRNA closely related to AD is E230001N04Rik, which was found to regulate the production of tau aggregates in an in vitro AD model.^20^ Even though many recent studies have demonstarted association of several lncRNAs with AD, the regulatory role of lncRNAs as ceRNAs in AD is not well characterized and needs further exploration.

Owing to the potential role of lncRNAs in the diagnosis and treatment of AD, we used the AlzData database and GEO database for mining analysis of genes associated with AD. By convergent functional genomics (CFG) ranking and analysis of differentially expressed genes (DEGs), early genes significantly associated with AD before overt pathological changes were identified in different brain regions. Subsequently, functional annotation and enrichment analysis were performed. We identified miRNAs and lncRNAs that could bind these genes. A lncRNA‐related ceRNA regulatory network composed of AD potential biomarker genes was constructed based on the ceRNA hypothesis. Our aim was to construct lncRNA‐associated ceRNA regulatory networks composed of potential biomarkers for the early stage of AD development.

## 2 MATERIALS AND METHODS

### 2.1 Acquisition of cross‐platform normalized expression data of AD microarray profile

**FIGURE 1:**
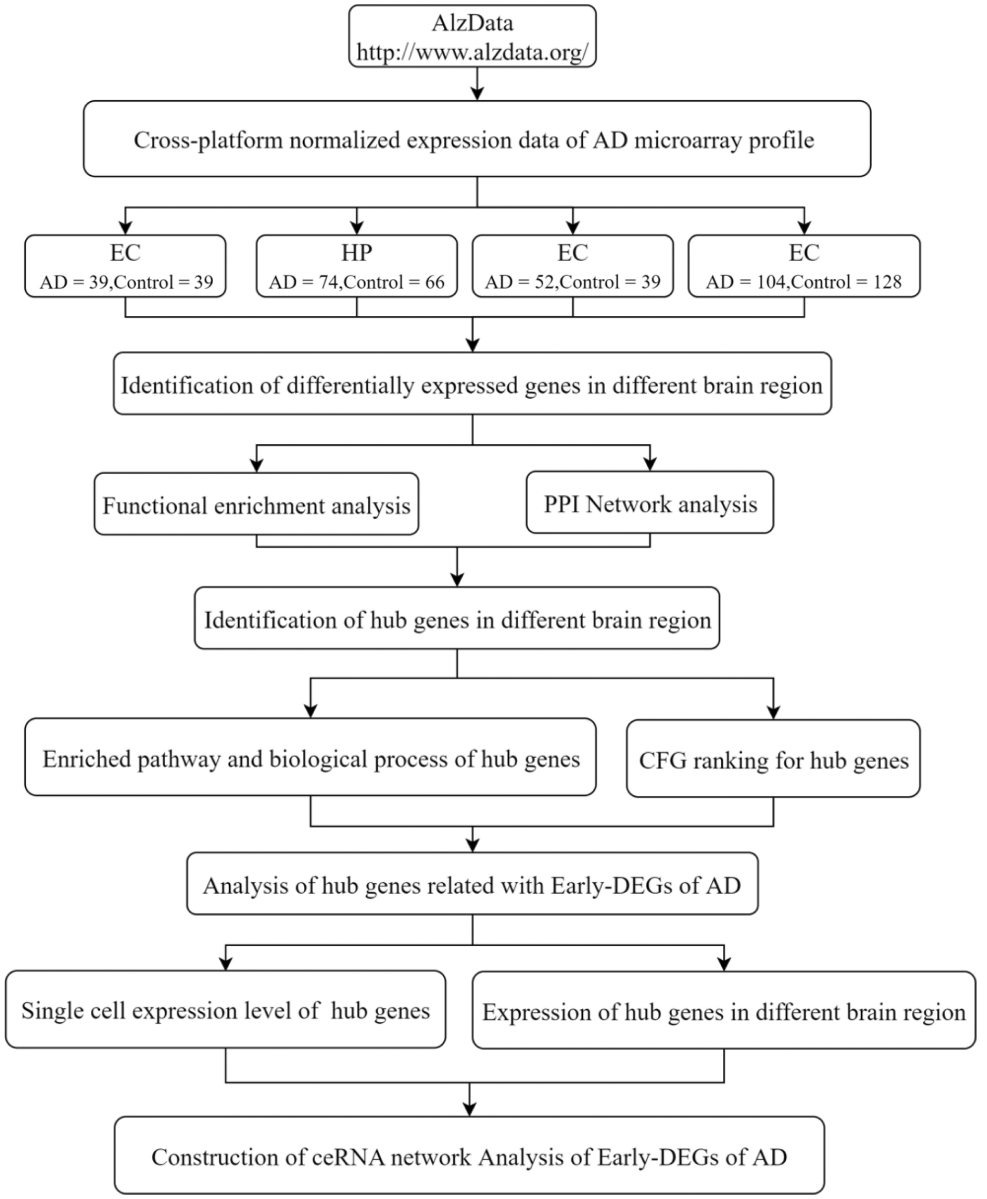
Flowchart illustrating the study design.

The research design is illustrated in the flowchart (Figure 1). We downloaded the cross‐platform normalized expression data of different brain regions related to AD in the Alzdata database (http://www.alzdata.org/),^21^, ^22^ including the entorhinal cortex (EC), hippocampus (HP), temporal cortex (TC) and frontal cortex (FC). Thirteen GEO datasets (GSE12685, GSE36980, GSE48350, GSE5281, GSE53890, GSE66333, GSE15222, GSE28146, GSE29378, GSE29652, GSE37263, GSE26927 and GSE26972) pertaining to a total of 541 brain samples (269 AD patients and 272 healthy individuals) were selected for further analysis (Table 1).

**TABLE 1:** Expression data characteristics.

	Brain region	Datasets	Sample	AD	Normal	AD		Normal	
						Female	Male	Female	Male
1	Entorhinal Cortex (EC)	GSE12685, GSE36980, GSE48350, GSE5281, GSE53890, GSE66333, GSE15222	78	39	39	18	21	17	22
2	Hippocampus (HP)	GSE28146, GSE29378, GSE36980, GSE48350, GSE5281	140	74	66	45	29	23	43
3	Temporal Cortex (TC)	GSE29652, GSE36980, GSE37263, GSE5281	91	52	39	14	20	18	21
4	Frontal Cortex (FC)	GSE26927, GSE26972, GSE48350, GSE5281	232	104	128	44	46	55	65

### 2.2 Identification of DEGs in different brain regions

Differential expression analysis was performed using the thresholds of |log2 (fold change) >0.05 and FDR (adjusted *p*‐value) <0.05 in EC, HP, TC. For FC, the thresholds of |log2 (fold change)| >0.03 and FDR (adjusted *p*‐value) <0.05 were used by Sangerbox^23^ (http://sangerbox.com/Index).

### 2.3 Function enrichment and protein–protein interaction (PPI) network analysis

We employed Metascape (https://metascape.org) to obtain information on DEGs in different brain regions for Gene Ontology (GO) analysis, including the involved biological processes (BP), cellular components (CC) and molecular functions (MF). Potential functions analysis was performed using the Kyoto Encyclopedia of Genes and Genomes (KEGG) pathway. *p* < 0.05 was set as the cutoff criteria and the plots were constructed using an online bioinformatics application (http://www.bioinformatics.com.cn). Using Cytoscape (version 3.6.0), we visualized the PPI network for DEGs in different brain regions using the STRING database.

### 2.4 Gene cluster identification and enriched pathway and biological processes of hub genes

MCODE in Cytoscape was employed to identify remarkable clusters in the PPI network. We selected cluster 1 of each PPI network in different brain regions as the hub genes. Then, KEGG enrichment pathways and BP of hub genes for each brain region were obtained through Metascape and visualized through bioinformatics tools.

### 2.5 Analysis of hub genes related to early‐DEGs of AD

In the Alzdata database, a CFG was conducted on hub genes that were differentially expressed in AD mouse models prior to the development of overt AD pathology (referred to as early‐DEGs). These early‐DEGs were visualized in Cytoscape (version 3.6.0) software. Then, Metscape was used to further analyse the relevant pathways and biological processes of early‐DEGs.

### 2.6 Prediction of pivotal miRNAs and construction of miRNA‐gene interaction networks

The miRNAs related to the crucial pathways targeted by Early‐DEGs were selected by miRWalk 2.0. The three databases of miRWalk, miRNet and miRTarBase were used to verify the accuracy of the intersection results. The final results were processed with Cytoscape after obtaining the intersection and identifying miRNAs that targeted more than two genes for further analysis.

### 2.7 Construction of lncRNA‐miRNA‐mRNA competing endogenous RNA network for analysis of early‐DEGs

Competing endogenous RNAs (ceRNAs) were predicted according to the hypothesis, and a lncRNA‐miRNA‐mRNA network related to early‐DEGs of AD was constructed. First, the miRNA expression profile and sample details of GSE16759 were downloaded from the publicly available repository GEO database (https://www.ncbi.nlm.nih.gov/geo/) to identify different expressions of miRNAs. Then, we used miRNet to predict lncRNAs. Eventually, a lncRNA‐miRNA‐mRNA network was established using Cytoscape.

### 2.8 Statistical analysis

R software (Version 3.6.3) was used for statistical analysis. The differences of gene data between two groups were compared by independent *t*‐test in 4 different brain regions. *p*‐values <0.05 were considered indicative of statistical significance.

## 3 RESULTS

### 3.1 Identification of DEGs in different brain regions

**FIGURE 2:**
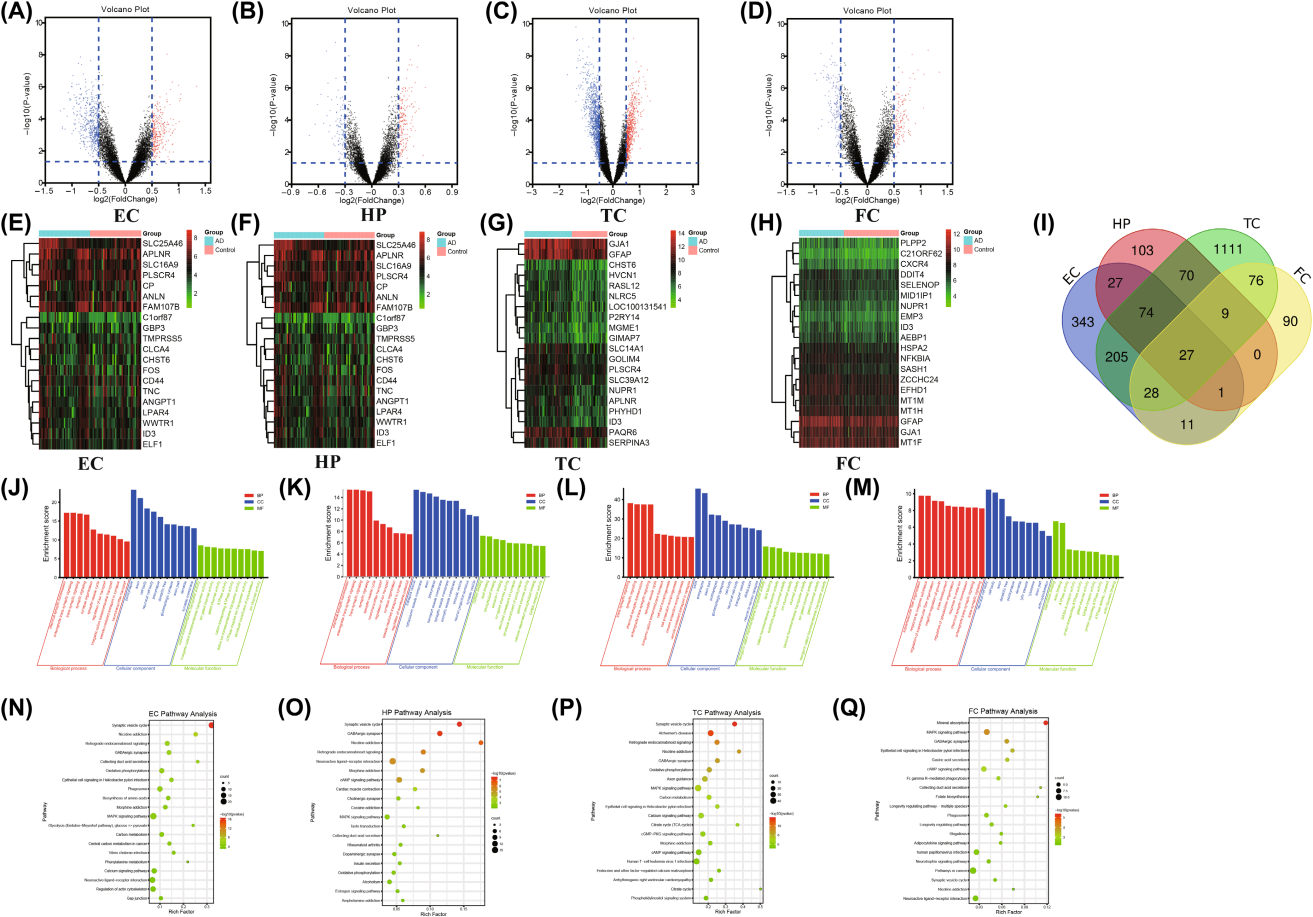
Identification of DEGs and functional enrichment analysis. Volcano plots showing the differentially expressed genes in AD and control samples of different brain regions. Blue dots represent significantly down‐regulated genes in AD samples and red dots represent significantly up‐regulated genes (A–D). Heatmap showing the expression levels of the top 20 DEGs in each dataset (E–H). Venn diagrams showing 29 DEGs (I) from datasets of 4 different brain regions. GO terms (J–M) and Top 20 enriched KEGG pathways (N–Q) are significantly associated with the expression of DEGs in 4 regions of the human AD brain.

We identified DEGs in different brain regions of AD patients and healthy individuals by downloading cross‐platform normalized expression data from the Alzdata database, including a total of 13 GEO datasets (GSE12685, GSE36980, GSE48350, GSE5281, GSE53890, GSE66333, GSE15222, GSE28146, GSE29378, GSE29652, GSE3726, GSE26927 and GSE26972). Among them, 716 genes (243 up‐regulated genes and 473 down‐regulated genes) for EC, 311 genes (137 up‐regulated genes and 174 down‐regulated genes) for HP, 1600 genes (568 up‐regulated genes and 1032 down‐regulated genes) for TC and 242 genes (148 up‐regulated genes and 94 down‐regulated genes) for FC were identified as DEGs (Table 2). Sangerbox and bioinformatics results were visualized with volcano plots (Figure 2A–D) and heatmaps (Figure 2E–H) according to gene expression analysis. OmicStudio tool (https://www.omstudio.cn/tool) was used to obtain intersecting DEGs of the 13 datasets, leading to the identification of 27 DEGs for each brain region (Figure 2I).

**TABLE 2:** DEGs in different brain regions.

	Brain region	AD vs Control		
		DEGs	Up‐regulated	Down‐regulated
1	Entorhinal Cortex (EC)	716	243	473
2	Hippocampus (HP)	311	137	174
3	Temporal Cortex (TC)	1600	568	1032
4	Frontal Cortex (FC)	242	148	94

### 3.2 Functional enrichment analysis of DEGs

Metascape was used to analyse GO terms and KEGG pathway enrichment of DEGs in four different brain regions (cutoff value *p* < 0.05). GO term enrichment analysis verified that DEGs in EC and FC brain regions were primarily related to the components of the neuronal body (Figure 2J,M), while those in the HP region were mostly associated with transport vesicles and chemical synaptic transmission (Figure 2K). DEGs in the TC region were mostly related to the component of the major axon (Figure 2L). The most important pathway of DEGs in EC, HP and TC brain regions revealed by KEGG enrichment analysis was synaptic vesicle cycle (Figure 2N–P), while genes in FC brain region were mainly involved in mineral absorption (Figure 2Q).

### 3.3 PPI network construction and hub genes analysis in four brain regions

**FIGURE 3:**
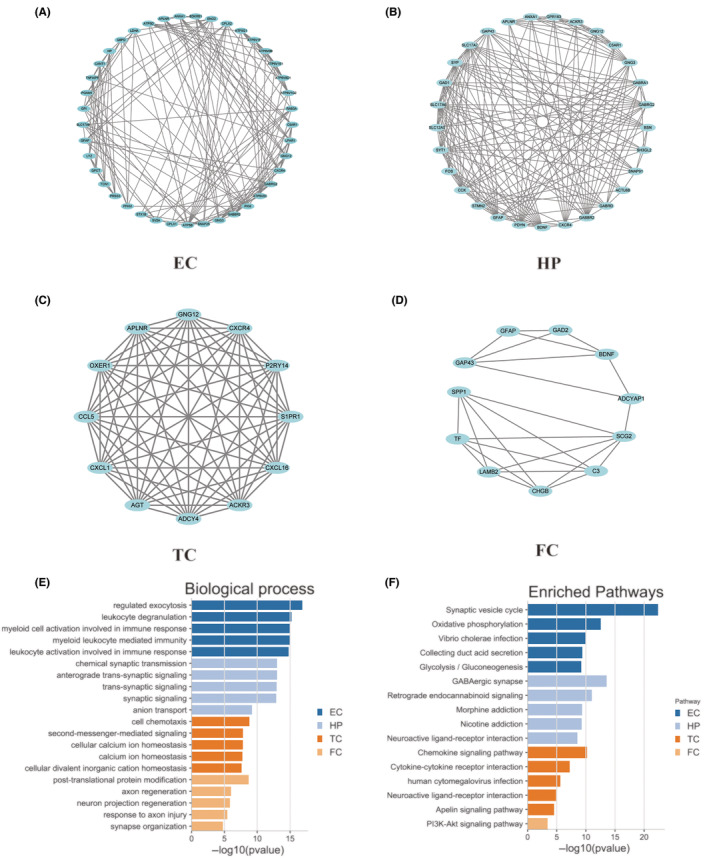
Analysis of key pathways and biological processes of hub genes in the PPI network. 41 hub genes in the EC region (A); 29 hub genes in the HP region (B); 12 hub genes in the TC region (C); and 11 hub genes in the FC region (D). The lines indicate the interaction of genes. Biological process (E) and enriched Top 5 pathways (F) of hub genes in different brain regions.

Using the STRING database, a PPI network was visualized for DEGs in different brain regions using Cytoscape. MCODE in Cytoscape was used to identify remarkable clusters in this PPI network. We selected cluster 1 in each PPI network of different brain regions as hub genes and screened out 41 genes in the EC region, 29 genes in the HP region, 12 genes in the TC region and 11 genes in the FC region for inclusion in the PPI network of the DEGs (Table 3, Figure 3A–D).

**TABLE 3:** Hub genes in different brain regions.

	Brain region	Count	Hub genes in cluster1 by MCODE
1	EC	41	*ANXA1*, *STX1B*, *GABBR2*, *TCN1*, *GABRG2*, *ATP6V1G2*, *PRSS3*, *ATP6V0B*, *CXCR4*, *ATP5G1*, *TNFAIP6*, *C5AR1*, *PGAM1*, *ENO2*, *GFAP*, *QPCT*, *SV2A*, *GNG12*, *CPLX2*, *SNAP25*, *PKM*, *CPLX1*, *ATP6V1E1*, *APLNR*, *ATP6V1F*, *LPAR1*, *SLC17A6*, *ATP6V0D1*, *ATP6V0C*, *LYZ*, *HP*, *RAB3A*, *CANT1*, *LDHA*, *ATP5D*, *ATP5B*, *GNG3*, *PFKM*, *BDKRB1*, *G6PD*, *GPI*
2	HP	29	*SLC12A5*, *ACKR3*, *STMN2*, *GFAP*, *ACTL6B*, *BDNF*, *GPR183*, *ANXA1*, *CXCR4*, *SNAP91*, *BSN*, *GABBR2*, *GAP43*, *PDYN*, *SH3GL2*, *SLC17A6*, *SLC17A7*, *GNG3*, *GABRA1*, *GABRG2*, *SYT1*, *GABRD*, *GAD1*, *FOS*, *GNG12*, *SYP*, *C5AR1*, *APLNR*, *CCK*
3	TC	12	*ADCY4*, *CXCL16*, *GNG12*, *P2RY14*, *AGT*, *CXCL1*, *OXER1*, *CCL5*, *APLNR*, *CXCR4*, *S1PR1*, *ACKR3*
4	FC	11	*ADCYAP1*, *BDNF*, *GAP43*, *GAD2*, *SPP1*, *C3*, *TF*, *GFAP*, *LAMB2*, *SCG2*, *CHGB*

### 3.4 Biological process analysis and crucial pathway verification of hub genes

Functional enrichment analysis of significant pathways and biological processes was performed on hub genes in 4 brain regions by Metascape (*p* < 0.05) (Tables S1 and S2). The results of BP enrichment revealed that the hub genes in the EC region were primarily involved in regulating exocytosis, and those in the HP region were mainly associated with chemical synaptic transmission. The hub genes in the TC region and FC region were involved in cell chemotaxis and post‐translation protein modification (Figure 3E). Besides, KEGG pathway enrichment of hub genes in EC region demonstrated the pathway that was primarily correlated to synaptic vesicle cycle. HP region was chiefly related to GABAergic synaptic pathway. In contrast, TC and FC regions were related to chemokine signalling and the PI3K‐Akt signalling pathway, respectively (Figure 3F).

### 3.5 CFG, biological process and pathway analysis of early‐DEGs in AD

**FIGURE 4:**
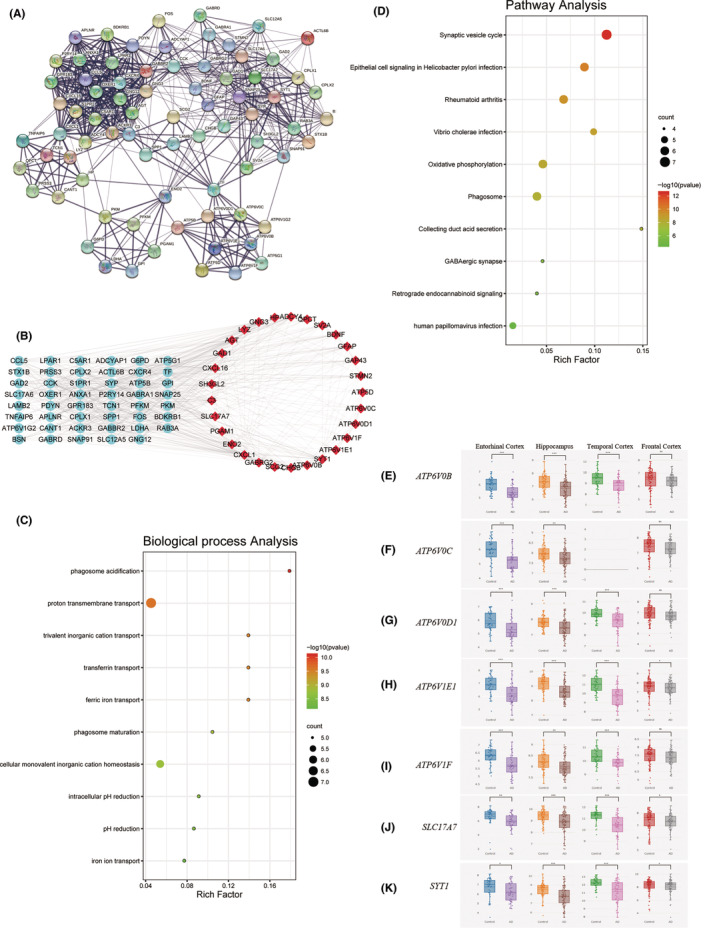
Analysis of pivotal pathways and biological process of Early‐DEGs and Cross‐platform normalized expression level of seven hub genes in different brain regions. The PPI interaction network of differentially expressed genes constructed in STRING database (A). Red represents the hub genes that were differentially expressed in AD mouse models before the emergence of AD pathology (B). Biological process (C) and pathway analysis (D) of hub genes that were differentially expressed in AD mouse models before the emergence of AD pathology. The expression levels of seven hub genes (*Atp6v0b*, *Atp6v0c*, *Atp6v0d1*, *Atp6v1e1*, *Atp6v1f*, *Slc17a7*, and*Syt1*) in different brain regions (E–K).

Hub genes were selected from cluster 1 of each PPI network involved in different brain regions (Figure 4A). To further testify the association of chosen hub genes with AD, CFG rank in Alzdata was used to analyse the 29 hub genes differentially expressed in the AD mouse model before the occurrence of AD pathology (early‐DEGs) and these early‐DEGs were visualized in Cytoscape software (Figure 4B). It was possible to effectively comprehend whether the expression level of hub genes was regulated by AD genetic variation. Hub genes associated with APP, PSEN1, PSEN2, APOE or MAPT interactions were significantly involved in AD pathology in Aβ or tau line AD mouse models. The results of CFG rank revealed that two genes (*GABRG2*, *GNG3*) in EC region, three genes (*GABRG2*, *GNG3*, *SH3GL2*) in HP region, two genes (*AGT*, *CXCL16*) in TC region and 1 gene in FC region (*C3*) were related with AD, among which *GABRG2*, *CXCL16* and *C3* were strongly correlated with Aβ and tau (Table S3). Moreover, the BP involved in hub genes was predominantly enriched in phagosome acidification and proton transmembrane transport (Figure 4C). Pathway enrichment results exhibited that the synaptic vesicle cycle was the dominant pathway concerned with hub genes (Figure 4D).

### 3.6 Further exploration of hub genes correlated to early‐DEGs of AD

Based on the results of pathway enrichment, seven genes (*Atp6v0b*, *Atp6v0c*, *Atp6v0d1*, *Atp6v1e1*, *Atp6v1f*, *Slc17a7* and *Syt1*) associated with the synaptic vesicle cycle were selected in each of the four brain regions (EC, HP, TC and FC) (Table 4). We examined the expression levels of hub genes by using the Alzdata. Only *ATP6V1E*, *Slc17a7* and *Syt1* were significantly differentially expressed (Figure 4H,J,K).

**TABLE 4:** Expression of seven hub genes in different brain regions.

Gene	Entorhinal Cortex			Hippocampus			Temporal Cortex			Frontal Cortex		
	logFC	*p*‐Value	FDR	logFC	*p*‐Value	FDR	logFC	*p*‐Value	FDR	logFC	*p*‐Value	FDR
*ATP6V0B*	−0.63	1.61E‐05	0.002	−0.51	9.45E‐06	0.002	−0.66	6.18E‐05	0.001	−0.05	0.533	0.684
*ATP6V0C*	−0.77	2.42E‐05	0.002	−0.22	0.002	0.034	NA	NA	NA	−0.11	0.138	0.278
*ATP6V0D1*	−0.65	1.73E‐04	0.006	−0.33	4.98E‐04	0.013	−0.64	4.47E‐05	0.001	−0.14	0.114	0.245
*ATP6V1E1*	−0.68	1.37E‐04	0.006	−0.52	7.89E‐07	0.001	−1.31	3.20E‐09	5.91E‐06	−0.23	0.012	0.056
*ATP6V1F*	−0.65	1.41E‐05	0.002	−0.23	0.002	0.026	−0.47	2.59E‐04	0.004	−0.09	0.151	0.294
*SLC17A7*	−0.9	0.001	0.018	−0.52	6.51E‐05	0.004	−0.84	6.59E‐07	8.64E‐05	−0.22	0.023	0.085
*SYT1*	−0.59	0.011	0.064	−0.67	2.92E‐05	0.003	−1.09	8.60E‐06	3.82E‐04	−0.33	0.02	0.077

### 3.7 Construction of ceRNA network analysis of early‐DEGs of AD

**FIGURE 5:**
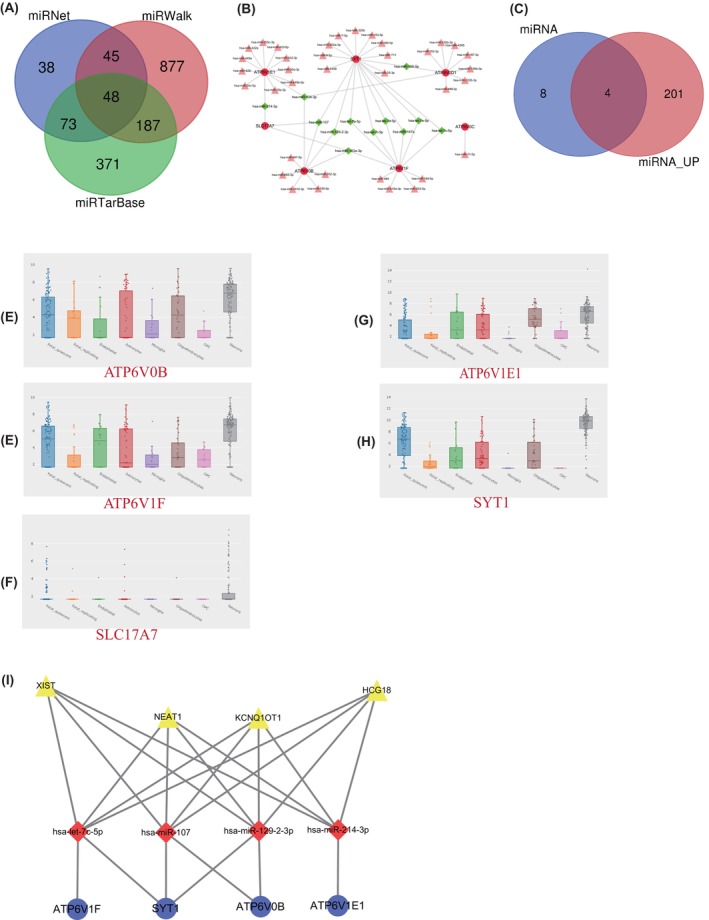
Hub gene expression and CeRNA regulatory network analysis. Venn diagrams showing the intersection of three databases of predicted miRNAs differentially expressed in the synaptic vesicle cycle pathway (A). miRNA‐mRNA network consisting of *Atp6v0b*, *Atp6v0c*, *Atp6v0d1*, *Atp6v1e1*, *Atp6v1f*, *Slc17a7 and Syt1* and their predicted miRNAs, respectively (B) Four miRNAs targeting *Atp6v0b*, *Atp6v1e1*, *Atp6v1f*, *Slc17a7* and *Syt1* were differentially expressed after cross‐expression with up‐regulated miRNAs and are shown in Venn diagrams (C). Differentially expressed miRNAs and their regulatory networks targeting mRNAs after intersection (D). Expression levels of five central genes (*Atp6v0b*, *Atp6v1e1*, *Atp6v1f*, *Slc17a7* and *Syt1*) in single brain cells (E–I). A regulatory network composed of LncRNA‐miRNA‐Mrna(J).

We predicted miRNA that targeted hub genes related to the synaptic vesicle cycle by performing miRWalk 2.0 (http://zmf.umm.uni‐heidelberg.de/mirwalk2). To verify the accuracy of the results, three databases, including miRWalk, miRNet and miRTarBase, were used for intersection analysis. Forty‐eight miRNAs obtained from the intersection were further processed and visualized with Cytoscape (Figure 5A). A total of 12 miRNAs targeting two or more genes were identified (Table S4, Figure 5B), among which 4 miRNAs targeting *Atp6v0b*, *Atp6v1e1*, *Atp6v1f*, *Slc17a7* and *Syt1* were differentially expressed after the intersection with up‐regulated miRNAs (Table S5, Figure 5C,D). Then, we further analysed the expression of *Atp6v0b*, *Atp6v1e1*, *Atp6v1f*, *Slc17a7* and *Syt1* at single cell level. The results manifested that in addition to *Slc17a7*, there were significantly differential expressions in brain cells of the other four genes (*Atp6v0b*, *Atp6v1e1*, *Atp6v1f* and *Syt1*) (Figure 5E–I). Based on the ceRNA hypothesis, lncRNA‐targeted hsa‐let‐7c‐5p, hsa‐miR‐107, hsa‐miR‐129‐2‐3p and hsa‐miR‐214‐3p were identified (Table S6). The lncRNA‐miRNA‐mRNA regulatory networks were constructed with 4 lncRNAs (HCG18 KCNQ1OT1 NEAT1 and XIST), 4 miRNAs (hsa‐let‐7c‐5p, hsa‐miR‐107, hsa‐miR‐129‐2‐3p and hsa‐miR‐214‐3p) and 4 mRNAs (*Atp6v0b*, *Atp6v1e1*, *Atp6v1f and Syt1*) (Figure 5J).

## 4 DISCUSSION

By the time AD is diagnosed clinically, significant changes have already occurred in neurons across multiple brain regions. In recent years, many studies have reported the mechanism of delaying the occurrence and development of neurodegenerative diseases. During cellular stress response, the cytoprotective genes of vitagenes produce molecules with antioxidant and anti‐apoptotic activities such as heme oxygenase‐1. (HO‐1), heat shock protein Hsp72, sirtuins and thioredoxin/thioredoxin reductase system are potential ways to prevent the development of neurodegenerative diseases.^24^, ^25^, ^26^, ^27^, ^28^, ^29^, ^30^ In addition, NO can also indirectly It confers cytoprotective effects by inhibiting caspase activity,^31^, ^32^ and promotes the overexpression of heme oxygenase (HO‐1) and the increase of biliverdin to exert neuroprotective functions.^33^, ^34^, ^35^ Concetta et al. also found that curcumin and 3,4‐dihydroxyphenylacetaldehyde (DOPAL) can also exert neuroprotective effects through the vitagenes system.^36^, ^37^ However, in the early stages of the disease, patients usually have no symptoms, but pathological changes have occurred in the nervous system. Therefore, identifying effective early biomarkers is very important to promote the clinical diagnosis and treatment of AD. In the classic symptom classification of AD, mild cognitive impairment (MCI) serves as a transitional state between normal ageing and AD. Research by Casati et al. found that TREM2 can be used as a peripheral biomarker for early AD (MCI).^38^ In addition, proteins such as NEL‐like protein 1, human kallikrein 14 and centrin‐2 can be detected in the early stages of AD and appear throughout the disease progression.^39^ This study constructed an interrelated ceRNA regulatory network by applying bioinformatics methods to investigate the key lncRNAs and miRNAs interacting with early‐DEGs of AD. The objective was to elucidate the potential early molecular and biological targets based on this ceRNA network. Four genes (*Atp6v0b*, *Atp6v1e1*, *Atp6v1f and Syt1*) were identified based on the bioinformatics analysis. They were significantly associated with the synaptic vesicle cycle during the early stages of the development of AD. Based on this finding, we discovered four lncRNAs (HCG18, KCNQ1OT1, NEAT1 and XIST) and four miRNAs (hsa‐let‐7c‐5p, hsa‐miR‐107, hsa‐miR‐129‐2‐3p and hsa‐miR‐214‐3p) and jointly constructed a ceRNA network. These markers provide new ideas for preventing the occurrence and development of AD in the early stages.

Our bioinformatics results suggest that the expression of *Syt1* was decreased in the four brain regions in AD patients. This is consistent with the previously reported downregulation of *Syt1* in multiple brain regions of AD patients.^40^, ^41^ SYT is an integral membrane protein of synaptic vesicles and is believed to act as a sensor of calcium ions (Ca^2+^) during vesicle trafficking and exocytosis. Among the known isoforms, the low‐affinity vesicular synaptotagmin *Syt1* functions in synchronized vesicle fusion.^42^ *Syt1* has also been shown to play a crucial role in the release of neurotransmitters in the presynaptic terminal ganglia.^43^ CFG analysis showed that *Syt1* can interact with MAPT, which is the coding gene of AD core Tau protein that is expressed in most nerve cells. This demonstrates a potential association of *Syt1* with the pathogenesis of AD. Besides, to the best of our knowledge, this is the first study reporting a potential role of *Atp6v0b*, *Atp6v1e1* and *Atp6v1f* in AD.

Benefiting from the relatively high stability of miRNA and its easy detection in body fluids, it has become an attractive biomarker for early AD. Studies have shown that miR‐191, miR‐103, miR‐125b, miR‐222 and miR‐193b are dysregulated in blood samples of AD patients and can be used as potential biomarkers for early AD.^44^, ^45^, ^46^, ^47^, ^48^, ^49^ Our results found that X‐inactivity specific transcript (XIST) can also target hsa‐let‐7c‐5p, hsa‐miR‐107, hsa‐miR‐129‐2‐3p and hsa‐miR‐214‐3p to affect the expressions of *Syt1*, *Atp6v0b*, *Atp6v1e1* and *Atp6v1f*. Several recent studies have reported the involvement of lncRNAs as ceRNAs in the pathogenesis of AD. XIST has been suggested to be mainly responsible for the inactivation of X chromosome and likely plays a role in the prevention of cerebral ischemic injury.^50^, ^51^ Furthermore, Wang et al. reported significant up‐regulation of lncRNA XIST in AD brain and that XIST induced the toxicity of Aβ to hippocampal neurons by targeting miR‐132.^52^ This is further supplemented by the 4 miRNAs identified in the present study. Our results are also partially consistent with previous studies in which another lncRNA (i.e. NEAT1) was found to regulate the development of AD by down‐regulating micro‐27a‐3p^53^ and targeting miR‐107 in AD was found to exacerbate Aβ‐induced neuronal damage.^54^ Besides, we also discovered three new miRNA targets. Although KCNQ1OT1 has been reported to be up‐regulated in in vitro matured (IVM) mouse offspring,^55^ to the best of our knowledge, this is the first study reporting that HCG18 and KCNQ1OT1 act as ceRNA regulators in the pathogenesis of AD. However, the specific mechanisms by which they affect AD remains unclear. In this study, we discovered more miRNA targets of NEAT1 and XIST, providing more insights for the diagnosis and treatment of early AD. Importantly, HCG18 and KCNQ1OT1 are potential candidate biomarkers for AD pathogenesis.

Most of the relevant contemporary literature pertains to studies exploring how lncRNAs participate in the pathogenesis of AD as ceRNA after pathological changes in AD. Zhang et al.^56^ identified three lncRNAs (AP000265.1, KB‐1460A1.5 and RP11‐145M9.4) as participating in the occurrence of NFTs by competing with miRNAs for binding. lncRNAs TCONS_00367775, TCONS_00323331 and TCONS_00204925 regulate miRNAs closely associated with Aβ, thereby inhibiting the expression of related mRNAs.^57^ The present study is the first study to analyse the early ceRNA network that may exist before the development of overt pathological changes of AD. The analysis of ceRNA regulatory network showed that lncRNA NEAT1, XIST, HCG18 and KCNQ1OT1 may competitively bind hsa‐let‐7c‐5p, hsa‐miR‐107, hsa‐miR‐129‐2‐3p and hsa‐miR‐214‐3p and regulate *Atp6v0b*, *Atp6v1e1*, *Atp6v1f* and *Syt1* in AD brain. Our results deepen the understanding of the underlying mechanism by which these lncRNAs regulate the expression of *Atp6v0b*, *Atp6v1e1*, *Atp6v1f* and *Syt1* and offer new ideas for the early prevention of AD. One of the limitations of the current study is that bioinformatics analysis was not validated by experimental techniques; their functions in causing AD require further investigation.

## 5 CONCLUSION

This study identifies genes that are significantly associated with early AD and searched for miRNAs and lncRNAs targeted genes. Four lncRNAs (XIST, NEAT1, KCNQ1OT1 and HCG18) and four miRNAs (hsa‐let‐7c‐5p, hsa‐miR‐107, hsa‐miR‐129‐2‐3p and hsa‐miR‐214‐ 3p) were identified as potential biomarkers for AD. Therefore, inhibiting endogenous XIST, NEAT1, KCNQ1OT1 and HCG18 expression are potential strategies for the prevention and treatment of AD. Moreover, our findings deepen the understanding of the potential role of ceRNAs in the early diagnosis and treatment of AD and provide new insights into the pathogenesis of AD.

## AUTHOR CONTRIBUTIONS

**Bin Huang:** Writing – original draft (equal); writing – review and editing (equal). **Guan‐yong Ou:** Data curation (equal); formal analysis (equal); writing – review and editing (equal). **Ni Zhang:** Data curation (equal); formal analysis (equal); writing – review and editing (equal).

## CONFLICT OF INTEREST STATEMENT

The authors declare that the research was conducted in the absence of any commercial or financial relationships that could be construed as a potential conflict of interest. The authors declare no conflict of interest.

## DATA AVILABILITY STATEMENT

The data that support the findings of this study are openly available in Alzdata database at http://www.alzdata.org/.

## Supporting information


Tables S1–S6.
