# Supplementary material for: Identification of key regulatory molecules in the early development stage of Alzheimer's disease
Source: J Cell Mol Med. 2024 Mar 1;28(6):e18151. doi: 10.1111/jcmm.18151 (PMC10907834; doi:10.1111/jcmm.18151)
Supplement: Supplementary file 1 — Tables S1–S6. [file JCMM-28-e18151-s001.docx]

**Supplementary Information for**

**Identification of key regulatory molecules in the early development stage of Alzheimer’s disease**

**Table S1. Enriched GO biological processes of Hub genes in different brain regions.**

|  | GO term | Biological process | Count | P-value |
| --- | --- | --- | --- | --- |
| EC | GO:0045055  GO:0043299  GO:0002275  GO:0002444  GO:0002366  GO:0002263  GO:0043312  GO:0002283  GO:0002446  GO:0042119 | regulated exocytosis  leukocyte degranulation  myeloid cell activation involved in immune response  myeloid leukocyte mediated immunity  leukocyte activation involved in immune response  cell activation involved in immune response  neutrophil degranulation  neutrophil activation involved in immune response  neutrophil mediated immunity  neutrophil activation | 18  15  15  15  16  16  14  14  14  14 | 1.39865E-17  6.11648E-16  8.45677E-16  9.91647E-16  1.68871E-15  1.84108E-15  4.55919E-15  4.95981E-15  6.72367E-15  6.90985E-15 |
| HP | GO:0007268  GO:0098916  GO:0099537  GO:0099536  GO:0006820  GO:0099504  GO:0048488  GO:0140238  GO:0015800  GO:0036465 | chemical synaptic transmission  anterograde trans-synaptic signaling  trans-synaptic signaling  synaptic signaling  anion transport  synaptic vesicle cycle  synaptic vesicle endocytosis  presynaptic endocytosis  acidic amino acid transport  synaptic vesicle recycling | 13  13  13  13  10  7  5  5  5  5 | 9.65476E-14  9.65476E-14  1.11024E-13  1.29674E-13  6.25257E-10  1.20956E-09  5.15151E-09  5.15151E-09  1.02985E-08  1.27145E-08 |
| TC | GO:0060326  GO:0019932  GO:0006874  GO:0055074  GO:0072503  GO:0030595  GO:0072507  GO:0070098  GO:0030335  GO:1990868 | cell chemotaxis  second-messenger-mediated signaling  cellular calcium ion homeostasis  calcium ion homeostasis  cellular divalent inorganic cation homeostasis  leukocyte chemotaxis  divalent inorganic cation homeostasis  chemokine-mediated signaling pathway  positive regulation of cell migration  response to chemokine | 6  6  6  6  6  5  6  4  6  4 | 1.59557E-09  1.53753E-08  1.55769E-08  1.81727E-08  2.44458E-08  2.88987E-08  3.05985E-08  4.42597E-08  5.6023E-08  6.56278E-08 |
| FC | GO:0043687  GO:0031103  GO:0031102  GO:0048678  GO:0050808  GO:0007409  GO:0061564  GO:0031099  GO:0048667  GO:0006935 | post-translational protein modification  axon regeneration  neuron projection regeneration  response to axon injury  synapse organization  axonogenesis  axon development  regeneration  cell morphogenesis involved in neuron differentiation  chemotaxis | 6  3  3  3  4  4  4  3  4  4 | 2.0285E-09  9.86996E-07  1.44969E-06  3.64733E-06  1.70928E-05  2.60235E-05  3.77187E-05  5.76706E-05  6.27622E-05  8.64827E-05 |

**Table S2. Enriched KEGG Pathway of Hub genes in different brain regions.**

|  | GO term | Biological process | Count | P-value |
| --- | --- | --- | --- | --- |
| EC | ko04721  ko00190  ko05110  hsa04966  ko00010  ko05120  ko05323  hsa01200  ko05230  ko04145 | Synaptic vesicle cycle  Oxidative phosphorylation  Vibrio cholerae infection  Collecting duct acid secretion  Glycolysis / Gluconeogenesis  Epithelial cell signaling in Helicobacter pylori infection  Rheumatoid arthritis  Carbon metabolism  Central carbon metabolism in cancer  Phagosome | 12  9  6  5  6  6  6  6  5  6 | 4.16079E-23  2.88714E-13  1.15499E-10  4.12584E-10  6.29118E-10  6.8926E-10  3.82931E-09  1.59854E-08  4.05279E-08  9.63918E-08 |
| HP | ko04727  ko04723  ko05032  ko05033  hsa04080  ko04724  ko05034  ko04721  hsa05170  hsa04713 | GABAergic synapse  Retrograde endocannabinoid signaling  Morphine addiction  Nicotine addiction  Neuroactive ligand-receptor interaction  Glutamatergic synapse  Alcoholism  Synaptic vesicle cycle  human immunodeficiency virus 1 infection  circadian entrainment | 8  7  6  5  8  4  4  3  4  3 | 2.79873E-14  9.41989E-12  4.46375E-10  5.32142E-10  2.90915E-09  5.72654E-06  3.46258E-05  3.80656E-05  8.8222E-05  0.00015106 |
| TC | ko04062  ko04060  hsa05163  hsa04080  hsa04371  hsa05200 | Chemokine signaling pathway  Cytokine-cytokine receptor interaction  human cytomegalovirus infection  Neuroactive ligand-receptor interaction  Apelin signaling pathway  Pathways in cancer | 6  5  4  4  3  4 | 6.21875E-11  6.02743E-08  2.47104E-06  1.36678E-05  2.9537E-05  7.64775E-05 |
| FC | hsa04151 | PI3K-Akt signaling pathway | 3 | 0.000377622 |

**Table S3. Convergent functional genomic (CFG) Ranks for Hub genes related with Early-DEGs in different brain regions.**

|  | Gene | eQTL | GWAS | PPI | Early_DEG | Pathology cor (abeta) | Pathology cor (tau) | CFG |
| --- | --- | --- | --- | --- | --- | --- | --- | --- |
| EC | GABRG2  GNG3  ATP6V0C  ENO2  GFAP  HP  LYZ  QPCT  SV2A  ATP5D  ATP6V0B  ATP6V0D1  ATP6V1E1  PGAM1  ATP6V1F | 1  1  9  2  0  0  0  NA  2  0  3  0  0  0  0 | 3  NA  NA  0  0  0  0  5  0  0  0  0  0  0  0 | NA  APP  NA  NA  PSEN1,PSEN2  APOE  APP,APOE  NA  NA  NA  NA  NA  NA  NA  NA | yes  yes  yes  yes  yes  yes  yes  yes  yes  yes  yes  yes  yes  yes  yes | -0.441,**  -0.612,***  -0.483,***  -0.022,ns  0.889,***  0.263,ns  0.905,***  -0.461,**  -0.199,ns  -0.370,*  -0.134,ns  -0.385,**  -0.534,***  -0.429,**  0.219,ns | -0.763,***  -0.333,ns  -0.106,ns  -0.547,*  0.711,**  0.745,**  0.811,***  -0.272,ns  -0.746,**  0.065,ns  0.346,ns  0.055,ns  -0.316,ns  -0.729,**  0.498,ns | 4  4  3  3  3  3  3  3  3  2  2  2  2  2  1 |
| HP | GABRG2  GNG3  SH3GL2  BDNF  GAD1  GAP43  GFAP  SYT1  SLC17A7  STMN2 | 1  1  1  3  3  3  0  0  1  0 | 3  NA  0  0  0  0  0  7  0  0 | NA  APP  PSEN2  PSEN1,PSEN2,MAPT  NA  NA  PSEN1,PSEN2  MAPT  NA  NA | yes  yes  yes  yes  yes  yes  yes  yes  yes  yes | -0.441,**  -0.612,***  -0.365,*  -0.201,ns  -0.207,ns  -0.608,***  0.889,***  -0.209,ns  -0.040,ns  -0.662,*** | -0.763,***  -0.333,ns  -0.393,ns  -0.168,ns  -0.562,*  -0.446,ns  0.711,**  -0.471,ns  0.129,ns  -0.713,** | 4  4  4  3  3  3  3  3  2  2 |
| TC | AGT  CXCL16  ADCY4  CXCL1 | 1  3  1  0 | 0  0  0  0 | APP,PSEN1,APOE  APP,APOE  APP  APP,APOE | yes  yes  yes  yes | -0.359,*  0.901,***  0.230,ns  0.183,ns | 0.002,ns  0.818,***  -0.145,ns  0.663,** | 4  4  3  3 |
| FC | C3  BDNF  GAP43  GFAP  SCG2  CHGB | 2  3  3  0  1  2 | 0  0  0  0  0  0 | APP,PSEN1,PSEN2,MAPT  PSEN1,PSEN2,MAPT  NA  PSEN1,PSEN2  NA  NA | yes  yes  yes  yes  yes  yes | 0.850,***  -0.201,ns  -0.608,***  0.889,***  -0.348,*  -0.010,ns | 0.761,***  -0.168,ns  -0.446,ns  0.711,**  -0.711,**  0.029,ns | 4  3  3  3  3  2 |

**Table S4. Information on screened miRNAs and their target genes.**

|  | miRNA | Gene targeted by miRNA | Gene count |
| --- | --- | --- | --- |
| 1 | hsa-let-7a-5p | SYT1, ATP6V1F | 2 |
| 2 | hsa-let-7c-5p | SYT1, ATP6V1F | 2 |
| 3 | hsa-let-7d-5p | SYT1, ATP6V1F | 2 |
| 4 | hsa-let-7e-5p | SYT1, ATP6V1F | 2 |
| 5 | hsa-let-7i-5p | SYT1, ATP6V1F | 2 |
| 6 | hsa-miR-103a-3p | SLC17A7, ATP6V0B, ATP6V1F | 3 |
| 7 | hsa-miR-107 | SYT1, SLC17A7, ATP6V0B | 3 |
| 8 | hsa-miR-124-3p | ATP6V0B, ATP6V1E1, ATP6V0D1 | 3 |
| 9 | hsa-miR-129-2-3p | SYT1, ATP6V0B | 2 |
| 10 | hsa-miR-147a | SYT1, ATP6V1F | 2 |
| 11 | hsa-miR-186-5p | SYT1, ATP6V0D1 | 2 |
| 12 | hsa-miR-214-3p | SLC17A7, ATP6V1E1 | 2 |

**Table S5. Information on miRNAs after intersecting with differentially expressed miRNAs and their targeted mRNAs.**

| Upregulated miRNA | Gene targeted by upregulated miRNA |
| --- | --- |
| hsa-let-7c-5p | SYT1, ATP6V1F |
| hsa-miR-107 | SYT1, SLC17A7, ATP6V0B |
| hsa-miR-129-2-3p | SYT1, ATP6V0B |
| hsa-miR-214-3p | SLC17A7, ATP6V1E1 |

**Table S6. Information on the lncRNA-miRNA-mRNA regulatory network.**

| miRNA | LncRNA |
| --- | --- |
| hsa-let-7c-5p | **HCG18 KCNQ1OT1 NEAT1 XIST** ARHGAP27P1-BPTFP1-KPNA2P3 CARMN CDKN2B-AS1 DRAIC G2E3-AS1 HEIH HELLPAR HOXA11-AS IER3-AS1 IQCH-AS1 LINC00265 LINC00294 LINC00665 LINC00885 LINC00894 LINC00963 LINC01001 LINC01678 LINC01806 LINC01978 LINC02242 LINC02381 LINC02432 LMCD1-AS1 MEG8 MIR29B2CHG MIR99AHG MIRLET7BHG MUC20-OT1 NUTM2A-AS1 OIP5-AS1 OLMALINC RPARP-AS1 SLC9A3-AS1 SNHG12 SNHG16 SNHG4 STAG3L5P-PVRIG2P-PILRB THSD4-AS1 TMEM147-AS1 TMPO-AS1 TRG-AS1 TTC28-AS1 TTTY15 UBL7-AS1 VASH1-AS1 ZNF337-AS1 ZNF436-AS1 ZNF571-AS1 |
| hsa-miR-107 | **HCG18 KCNQ1OT1 NEAT1 XIST** ALMS1-IT1 ARHGAP11B CYTOR DLG1-AS1 DLX6-AS1 DUBR EPHA1-AS1 FAM30A FGD5-AS1 GUSBP11 H19 HOTAIRM1 LINC00294 LINC00630 LINC00638 LINC00641 LINC00662 LINC00707 LINC00960 LINC01134 LINC01343 LINC01569 LINC01848 LINC02035 LINC02434 LIPE-AS1 MEG8 MIR181A1HG MIR29B2CHG MIR503HG NFIA-AS2 NUTM2A-AS1 NUTM2B-AS1 PAX8-AS1 PCAT18 PCBP1-AS1 PSMA3-AS1 RNF139-AS1 RPARP-AS1 RRN3P2 SLC9A3-AS1 SNHG26 STAG3L5P-PVRIG2P-PILRB TEX41 TMEM147-AS1 TRPM2-AS TTC28-AS1 TTN-AS1 ZNF793-AS1 |
| hsa-miR-129-2-3p | **HCG18 KCNQ1OT1 NEAT1 XIST** ARHGAP27P1-BPTFP1-KPNA2P3 CEBPB-AS1 FGD5-AS1 GSN-AS1 HCG11 HCG9 KCTD21-AS1 LINC00511 LINC00886 LINC00958 LINC01006 LINC01343 MAPKAPK5-AS1 MIR4500HG NORAD NR2F2-AS1 OTUD6B-AS1 SATB2-AS1 SNHG1 SNHG12 TNK2-AS1 TP53TG1 VASH1-AS1 WEE2-AS1 |
| hsa-miR-214-3p | **HCG18 KCNQ1OT1 NEAT1 XIST** ADAMTSL4-AS1 ADIRF-AS1 ARHGAP11B ARMCX5-GPRASP2 ASB16-AS1 BACE1-AS C18orf15 C5orf66 DNAH17-AS1 DUXAP8 EPB41L4A-AS1 EXOC3-AS1 EXTL3-AS1 FENDRR FGD5-AS1 FRMD6-AS1 GACAT2 GAS1RR HCG17 HCP5 HEIH HOTAIR HOXA11-AS ITPKB-IT1 LINC00205 LINC00242 LINC00324 LINC00482 LINC00608 LINC00641 LINC00652 LINC00665 LINC00842 LINC00843 LINC00865 LINC00882 LINC00894 LINC00958 LINC01128 LINC01503 LINC01535 LINC01703 LINC01725 LINC01811 LINC02128 LINC02195 LINC02344 LOXL1-AS1 MAPT-IT1 MCM3AP-AS1 MIR17HG MIR193BHG MIR29B2CHG MUC20-OT1 NBR2 OLMALINC PCAT5 POLR2J4 PPP1R26-AS1 PRICKLE2-AS1 PSMA3-AS1 PVT1 RN7SL832P RUSC1-AS1 SCGB1B2P SDCBP2-AS1 SLC25A25-AS1 SLC9A3-AS1 SLFNL1-AS1 SLX1B-SULT1A4 SNHG14 SNHG16 SNHG17 SNHG3 SPINT1-AS1 STAG3L5P-PVRIG2P-PILRB TBX2-AS1 TMEM132D-AS1 TMEM147-AS1 TNFRSF14-AS1 TRIM52-AS1 TSPEAR-AS2 TYMSOS UCKL1-AS1 VASH1-AS1 VPS9D1-AS1 ZNF710-AS1 |
